# Basal forebrain activity predicts functional degeneration in the entorhinal cortex in Alzheimer’s disease

**DOI:** 10.1093/braincomms/fcad262

**Published:** 2023-10-09

**Authors:** Marthe Mieling, Martin Göttlich, Mushfa Yousuf, Nico Bunzeck

**Affiliations:** Department of Psychology, University of Lübeck, Lübeck 23562, Germany; Department of Neurology, University of Lübeck, Lübeck 23562, Germany; Center of Brain, Behavior and Metabolism, University of Lübeck, Lübeck 23562, Germany; Department of Psychology, University of Lübeck, Lübeck 23562, Germany; Department of Psychology, University of Lübeck, Lübeck 23562, Germany; Center of Brain, Behavior and Metabolism, University of Lübeck, Lübeck 23562, Germany

**Keywords:** Alzheimer’s disease, resting-state fMRI, fractional amplitude of low-frequency fluctuations (fALFF), basal forebrain, entorhinal cortex

## Abstract

Recent models of Alzheimer’s disease suggest the nucleus basalis of Meynert (NbM) as an early origin of structural degeneration followed by the entorhinal cortex (EC). However, the functional properties of NbM and EC regarding amyloid-β and hyperphosphorylated tau remain unclear. We analysed resting-state functional fMRI data with CSF assays from the Alzheimer’s Disease Neuroimaging Initiative (*n* = 71) at baseline and 2 years later. At baseline, local activity, as quantified by fractional amplitude of low-frequency fluctuations, differentiated between normal and abnormal CSF groups in the NbM but not EC. Further, NbM activity linearly decreased as a function of CSF ratio, resembling the disease status. Finally, NbM activity predicted the annual percentage signal change in EC, but not the reverse, independent from CSF ratio. Our findings give novel insights into the pathogenesis of Alzheimer’s disease by showing that local activity in NbM is affected by proteinopathology and predicts functional degeneration within the EC.

## Teaser

Mieling *et al*. report that local activity in the nucleus basalis of Meynert (NbM) differentiates between normal and abnormal CSF and decreases linearly with CSF ratio. NbM activity predicts signal changes in the entorhinal cortex, but not vice versa, independent of CSF ratio, offering novel insights into Alzheimer’s disease pathogenesis.

## Graphical Abstract

**Graphical Abstract:**
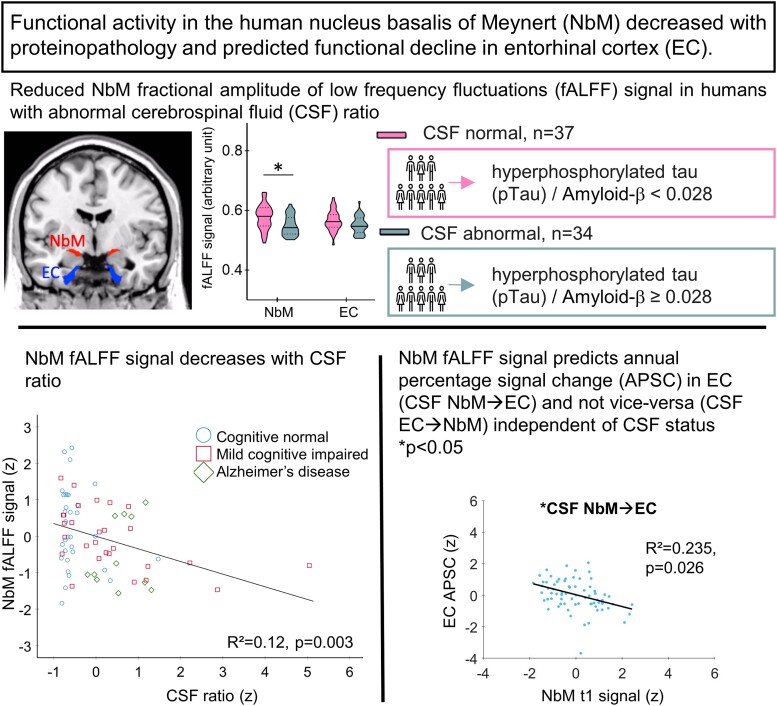

## Introduction

The basal forebrain’s nucleus basalis of Meynert (NbM) has recently been suggested as an early origin of structural degeneration in Alzheimer’s disease followed by the entorhinal cortex (EC) and other cortical brain regions.^1,2^ For instance, grey matter loss was more prominent in the NbM compared with the EC in cognitively healthy humans with an abnormal CSF biomarker of amyloid-β (Aβ) and hyperphosphorylated Tau (pTau).^2^ Moreover, the NbM’s baseline volume predicted the longitudinal structural degeneration in the EC, further suggesting a trans-synaptic spread of neuropathology starting in the NbM.^1,2^ This observation in humans is in line with animal work and adds a crucial upstream link to the subsequent spread from EC to other medial temporal lobe structures, including the hippocampus, and more distal neocortical brain regions such as the posterior parietal cortex.^1,3-6^ Importantly, evidence in favour of such a pathological staging model is mainly limited to anatomical studies, and, therefore, the functional properties of both the NbM and EC during the disease progression of Alzheimer’s disease in humans remain unclear.

Since functional brain changes in Alzheimer’s disease often precede structural degeneration,^7-9^ we investigated the functional properties of the NbM and EC, including their functional connectivity. To this end, we used data from the Alzheimer’s Disease Neuroimaging Initiative (ADNI) and performed a longitudinal region of interest (ROI) analysis over 2 years, focusing on regional and interregional resting-state functional MRI (rsfMRI) properties. In detail, we analysed (i) the fractional amplitude of low-frequency fluctuations (fALFF) to quantify spontaneous neuronal activity,^10-12^ (ii) regional homogeneity (ReHo) reflecting the synchronicity of neural activity between a voxel and its neighbouring voxels^13^ and finally (iii) the functional connectivity between NbM and EC. While all three measures may help to gain new insights into Alzheimer’s disease progression, we initially focused on fALFF given its established role^14-16^ and report ReHo and functional connectivity analyses in the Supplementary material.

In the first step, baseline signals and longitudinal functional changes were compared based on harmonized CSF assays of Aβ and pTau in NbM and EC. Subsequently, we investigated functional changes in disease progression using the CSF markers. Finally, we tested the competing models NbM→EC versus EC→NbM on a functional level. Our main hypothesis was that functional signals in the NbM predict functional changes in EC, which would provide further evidence supporting the pathological staging model from NbM to EC. From a more general perspective, we aimed to provide new insights into the underlying functional properties of Alzheimer’s disease, which may contribute to further developing markers and treatment strategies.

## Materials and methods

### ADNI data

Data used in the preparation of this article were obtained from the ADNI database (adni.loni.usc.edu). ADNI was launched in 2003 as a public–private partnership, led by Principal Investigator Michael W. Weiner, MD, to test whether serial MRI, PET, other biological markers and clinical and neuropsychological assessment can be combined to characterize the progression of mild cognitive impairment (MCI) and early Alzheimer’s disease.

Since rsfMRI was not acquired in all ADNI cohorts, here, data were combined from ADNI-GO, ADNI-2 (ADNI-GO/2) and ADNI 3, downloaded from the Image and Data Archive (IDA) platform run by the Laboratory of Neuro Imaging (LONI) (https://ida.loni.usc.edu). Specifically, we only selected data from participants with CSF biomarkers and two rsfMRI scans acquired with a delay of 2 years with the same MR scanner and head coil to ensure within-subject comparability.

### Image acquisition

Participants were scanned at multiple sites equipped with 3-Tesla MRI scanners according to unified ADNI monitoring protocols.^17^ To ensure maximum compatibility between the measurements, we followed ADNI’s recommendations and included only the basic rsfMRI version but not advanced version of ADNI 3 since it is not compatible with ADNI-GO/2. Moreover, all participants here were examined with the same scanner and head coil for both time points, *t*_1_ and *t*_2_ (https://adni.loni.usc.edu/methods/mri-tool/mri-analysis/). Further, we only included MRI data with excellent, good or fair quality. For further information on image acquisition, see the Supplementary material and http://adni.loni.usc.edu.

### Data preprocessing

Considering their specific scanning parameters such as TR, slice order and volume number, all data were preprocessed with the Data Processing Assistant for Resting-State fMRI Advanced (DPARSFA, http://rfmri.org/dpabi) toolbox version 5 (release 5.2_210501), which is based on the Statistical Parametric Mapping toolbox (SPM 12, https://www.fil.ion.ucl.ac.uk/spm/) for MATLAB^®^. It started with the removal of the first 10 volumes and subsequently included the following steps (i) slice time correction; (ii) spatial realignment; (iii) T_1_ co-registration to the mean functional image; (iv) CSF, grey and white matter tissue segmentation and spatial normalization using diffeomorphic anatomical registration using exponential lie algebra (DARTEL)^18^ for T_1_ images; (v) regression of nuisance variables; and (vi) normalization to the Montreal Neurological Institute (MNI) space and resampling to an isotropic voxel size of 3 mm of the functional images using the parameters estimated by DARTEL (see Supplementary material for a detailed description).

To reduce the influence of excessive head motion, participants exhibiting more than 3.0 mm of maximum movement and a 3.0-degree rotation angle were discarded. Further, images were visually inspected after co-registration, segmentation and normalization to guarantee high quality. This included a specific focus on signal loss and artefacts in our regions of interest (NbM, EC) by overlaying a ROI mask in standardized space; especially, the EC represents a region that might often be affected by artefacts.^19^ For a detailed description of the preprocessing steps, excluded participants, ROI definition and rsfMRI analyses for fALFF, ReHo and the functional connectivity, see Supplementary material.

### CSF biomarker

Alzheimer’s disease neuropathology includes the accumulation of Aβ resulting in plaques and pTau leading to neurofibrillary tangles.^20,21^ To better understand how both relate to functional degeneration in NbM and EC, we followed previous studies^1,2^ and used ADNI’s CSF samples, produced with a fully automated Elecsys^®^ protocol of Aβ and pTau from the first measurement (*t*_1_). For each participant, we extracted Aβ 1–42 and pTau181 values. Since the protocols by Elecsys^®^ are still under development, the results are restricted to a specific technical limit (>1700 pg/mL). Higher values were provided by extrapolation of the calibration curve for research purposes only but not diagnostics. Further information on CSF draws and analyses can be found at http://adni.loni.usc.edu.

Here, we analysed both proteins by using a previously established ratio of pTau/Aβ, which is known to highly concord with PET measures and clinical diagnoses.^22,23^ Based on these findings, the standardized and cross-validated cut-off of 0.028 was used to divide the participants into an abnormal (pTau/Aβ ≥ 0.028) and a normal (pTau/Aβ < 0.028) CSF group.^1,22,23^ Importantly, no participant classified with Alzheimer’s disease had a normal CSF ratio, but a few (*n* = 10) participants classified with MCI did, which indicates an unclear aetiology. Nevertheless, we included them based on biological instead of a syndromal grouping.^24^

### Neuropsychological assessment and clinical diagnosis

All participants underwent a comprehensive neuropsychological test battery. Here, *t*_1_ scores are used, including validated memory (MEM) and executive function (EF), based on a confirmatory factor analysis.^25,26^ Memory scores include the Alzheimer’s Disease Assessment Scale, Logical Memory test, Mini-Mental State Examination (MMSE) and Rey Auditory Verbal Learning Test (RAVLT). EF scores are based on the Category Fluency, Digit Span Backwards, Digit Symbol Substitution, Trails A and B and the Clock Drawing tests.^25,26^ We were also interested in the Montreal Cognitive Assessment (MoCA), Sum of Boxes in the Clinical Dementia Rating Scale (CDRSB) and the Alzheimer’s Disease Assessment Scale-Cognition Subscale, 13 tasks (ADAS-Cog 13) to get a deeper understanding of the participants’ cognitive profiles (see below).

Furthermore, we included participants’ *t*_1_ diagnosis made by the ADNI Clinical Core: cognitive normal (CN) [global Clinical Dementia Rating Scale (CDR) score = 0, MMSE = 24–30], MCI (global CDR score = 0.5, MMSE = 24–30) and Alzheimer’s disease (global CDR score = 0.5–1, MMSE = 20–26). These classifications represent widely used cognitive and functional measures in clinical trials.^27-29^ Further information regarding diagnostic is available at http://adni.loni.usc.edu.

### Participants

We included rsfMRI data from ADNI-GO/2 and ADNI 3—but, importantly, only those that also offered a subject’s CSF draw (see below) temporally related to a rsfMRI acquisition (e.g. a participant’s screening MRI and baseline lumbar punction measurement). This measurement served as *t*_1_ measurement in the analyses. To maximize the number of subjects, the second measurement was selected after an interval of 1.5 years ±12 months (*t*_2_).^1^ Further details on inclusion and exclusion criteria for participating in ADNI are available under http://adni.loni.usc.edu. In total, 153 participants for ADNI-GO/2 and 141 for ADNI 3 (only basic rsfMRI version) fulfilled our inclusion criteria. However, a large proportion had to be excluded mainly based on fMRI data quality (see Supplementary material). Thus, data from *n* = 71 participants were analysed, which could be further subdivided into those with normal CSF (nCSF, *n* = 37) and abnormal CSF (aCSF, *n* = 34) values (Table 1).

**Table 1:** Participants’ demographics and information on APOE4 genotype and harmonized CSF assays

	Normal CSF	Abnormal CSF	Test—*χ*²/*t*
*n* (total) = 71 ADNI-GO/2 (*n* = 44)/3 (*n* = 27)	37 17/20	34 27/7	*χ*² = 0.127, *P* = 0.722 *χ*² = 8.420, *P* = 0.004**
CN (*n* = 32)/MCI (*n* = 28)/AD (*n* = 11)	27/10/0	5/18/11	*χ*² = 28.335, *P* < 0.001***
Manufacturer Philips (*n* = 52)/Siemens (*n* = 11)/GE (*n* = 8)	24/8/5	28/3/3	*χ*² = 2.959, *P* = 0.228
Age	70.51 (6.23)	72.71 (7.18)	*t* = −1.376 *P* = 0.173
Female (*n* = 44)/male (*n* = 27)	22/15	22/12	*χ*² = 0.207, *P* = 0.649
Education (in years)	16.59 (2.44)	15.91 (2.25)	*t* = 1.222, *P* = 0.226
Interscan interval In months In days	22.03 (5.0) 685.35 (151.15)	18.74 (6.9) 587.76 (210.43)	*t* = 2.294 *P* = 0.025* t = 2.227, *P* = 0.030*
APOE4 (0/1/2)	28/8/1	5/21/8	*χ*² = 27.224, *P* < 0.001***
Aβ	1430.64 (521.29)	637.21 (187.10)	*t* = 8.670, *P* < 0.001***
pTau	18.42 (4.83)	40.81 (17.76)	*t* = −7.114, *P* < 0.001***

Information of the final sample from ADNI-GO/2 and ADNI-3 grouped by CSF. Means and standard deviation (SD) are represented and the respective *t*-test or *χ*² test to investigate possible group differences. Age and education were assessed in years. APOE4 status: no allele/1 allele/2 alleles. Aβ, amyloid-β in pg/mL as concentration of the amyloid-β 1–42 peptide. PTau, in pg/mL as CSF concentration of hyperphosphorylated tau.

Baseline clinical diagnosis: CN, cognitive normal; MCI, mild cognitive impairment; ad, Alzheimer’s disease.

*P < 0.05, **P < 0.01, ***P < 0.001.

Table 1 gives an overview of the participants’ demographics, as well as information on APOE4 genotype and harmonized CSF assay, and Table 2 shows the neuropsychological test results at baseline (*t*_1_).

**Table 2:** Neuropsychological test results at baseline, compared by CSF normal versus abnormal

Neuropsychological testing	CSF groups [mean (SD)]		*F*-value	*P*-value
	Normal	Abnormal		
MEM score	0.88 (0.6)	−0.08 (0.97)	*F*(1, 66) = 23.4	<0.001*
EF score	1.02 (0.76)	−0.16 (1.1)	*F*(1, 66) = 23.8	<0.001*
MMSE	29.08 (1.12)	25.82 (3.5)	*F*(1, 66) = 23.35	<0.001*
ADAS-Cog 13	9.8 (4.8)	22.89 (14.26)	*F*(1, 66) = 26.82	<0.001*
CDRSB	0.3 (0.55)	2.63 (2.36)	*F*(1, 66) = 34.73	<0.001*
MoCA	25.89 (2.34)	20.91 (5.72)	*F*(1, 66) = 22.5	<0.001*
Clock Drawing	4.76 (0.55)	4.03 (1.22)	*F*(1, 66) = 8.73	0.004*

The mean values with standard deviation (SD) for normal versus abnormal CSF groups. The abnormal CSF group showed worse performance in all neuropsychological tests.

MEM, memory function score; EF, executive function score; MMSE, Mini-Mental State Examination; ADAS-Cog 13, Alzheimer’s Disease Assessment Scale-Cognition Subscale, 13 tasks; CDRSB, Clinical Dementia Rating Scale Sum of Boxes; MoCA, Montreal Cognitive Assessment; Clock Drawing, Clock Drawing test.

*Significant after Bonferroni correction *P* < 0.05/*n* (*n* = 7 tests).

### Ethics approval and consent to participate

Each centre collecting data for ADNI provided an Institutional Review Board (IRB) approval and meets ADNI’s requirements. Informed consent was obtained from all ADNI participants (for more information at http://adni.loni.usc.edu). The analyses presented here were approved by the local Ethics Committee of the University of Lübeck and carried out after ADNI’s recommendations including the approval of the manuscript before submitting to a journal.

### Statistical analyses

#### Mixed ANCOVA

Mixed ANCOVAs were carried out for all measures separately (i.e. fALFF and ReHo) to compare baseline signals and the annual percentage signal change (APSC, see below) between regions (NbM and EC as a within-subject factor) and CSF groups (normal and abnormal as a between-subject factor). Covariates such as age, sex, education, ADNI cohort and scanner manufacturer were included to adjust for different scan protocols and other potential scanner-related differences. All 2 × 2 (region × CSF group) mixed ANCOVAs were carried out in IBM SPSS statistics version 25 (SPSS) with type III sums of squares, and within-subject effects were interpreted without covariates.^30^

#### Linear regression of disease status based on CSF marker

To better understand the relationship between disease status and functional MRI properties, CSF ratios (see section CSF biomarker) and functional MRI signals were considered in a linear regression model in SPSS. The functional MRI signal served as dependent variable and CSF ratio as independent variable. The regression was run with the *Z*-scored data. Subsequently, the dependent overlapping correlations of NbM versus EC with CSF ratio were compared using cocor.^31,32^

#### Robust regression

To minimize the influence of outliers, especially in the APSC, robust regression models were carried out in MATLAB^®^ R2020b with fitlm using the bisquare weight function with the default tuning constant. The same covariates as for the mixed ANCOVA were included in the model. Finally, the predictive models (NbM→EC and EC→NbM) were tested for each CSF group (normal and abnormal) and each functional property (fALFF and ReHo). The data were *Z*-scored before entering the analysis to ensure comparability of the APSC and baseline signal.

#### Moderation analyses of independent samples

Moderation analyses were carried out in SPSS using the PROCESS macro^33^ for fALFF and ReHo investigating whether CSF group assignment moderates the spread (NbM→EC versus EC→NbM) of functional degeneration. Here, CSF group was used as a dichotomous moderator variable. For the construction of products mean-centering was applied, and the heteroscedasticity consistent standard error HC3 (Davidson–MacKinnon) was applied.

### APSC

The following formula^1,34^ was used to assess longitudinal APSC in fALFF and ReHo. It accounts for the days between both measurements and minimizes the influence of differences between both measurements within a subject.


$$\begin{array}{rl} \mathrm{APSC}\;= & \left(\frac{\mathrm{Change}\;\mathrm{baseline}\;(t2-t1)\;\mathrm{signal})}{\mathrm{Baseline}\;\mathrm{signal}}\right)\\ & \times \left(\frac{365}{\mathrm{Interscan}\;\;\mathrm{interval}\;\;\mathrm{in}\;\;\mathrm{days}}\right)\times 100 \end{array}$$

## Results

### CSF grouping strategy and neuropsychological assessments

Based on the CSF grouping strategy, we investigated how abnormal CSF and normal CSF groups performed in neuropsychological tests. For each test, one-way fixed effect ANOVAs were carried out with CSF group as factor and age, sex and education as covariates. As expected, the normal CSF group is less affected by cognitive impairment than the abnormal CSF group (see Table 2).

### Lower fALFF values at baseline in abnormal CSF versus normal CSF in NbM but not EC

**Figure 1:**
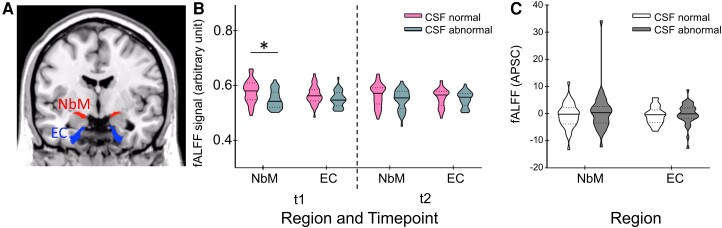
**Regions of interests (ROIs), baseline and follow-up signal and annual percentage signal change for fALFF**. (**A**) ROIs for the nucleus basalis of Meynert (NbM) and entorhinal cortex (EC) on a coronal slice of a T_1_-weighted standard brain template. Violin plots representing the participants’ (**B**) baseline fALFF signals at time point 1 (*t*_1_) and fALFF signals at the follow-up measurement (*t*_2_) for normal CSF (*n* = 37) and abnormal CSF (*n* = 34). A mixed ANCOVA revealed a main effect of CSF [*F*(1, 63) = 7.943, *P* = 0.006] and a significant region × CSF group interaction [*F*(1, 63) = 4.623, *P* = 0.035]. (**C**) Participants’ APSC in both regions again for normal CSF (*n* = 37) and abnormal CSF (*n* = 34). For the APSC, the mixed ANCOVA did not reveal significant effects. The horizontal lines represent medians and dotted lines interquartile ranges. *P < 0.01.

Baseline fALFF values were compared in NbM and EC further subdivided into CSF groups using a 2 × 2 mixed ANCOVA. We found a main effect of CSF group [*F*(1, 63) = 7.943, *P* = 0.006, *η*^2^_*p*_ = 0.112; Fig. 1B], which was driven by lower fALFF values in participants with abnormal CSF, and a significant region × CSF group interaction [*F*(1, 63) = 4.623, *P* = 0.035, *η*^2^_*p*_ = 0.068; Fig. 1B]. Post-hoc analyses showed that a significant difference in fALFF between normal CSF versus abnormal CSF was only observed in NbM [*t*(69) = 3.141, *P* = 0.002) but not EC (*t*(69) = 1.856, *P* = 0.068]. There was no main effect of region [*F*(1, 69) = 2.643, *P* = 0.109, *η*^2^_*p*_ = 0.037; Fig. 1B].

### APSC in fALFF does not differentiate between CSF groups or regions

We used a 2 × 2 mixed ANCOVA to investigate whether the longitudinal indices of APSC in fALFF of the NbM and EC differentiated between CSF normal versus abnormal groups. There was no significant main effect of CSF group [*F*(1, 63) = 2.077, *P* = 0.154, *η*^2^_*p*_ = 0.032; Fig. 1C] or region [*F*(1, 69) = 0.499, *P* = 0.482, *η*^2^_*p*_ = 0.007] and no significant group × region interaction [*F*(1, 63) = 0.367, *P* = 0.547, *η*^2^_*p*_ = 0.006] in APSC fALFF (Fig. 1C).

### NbM’s fALFF relates to CSF ratio

**Figure 2:**
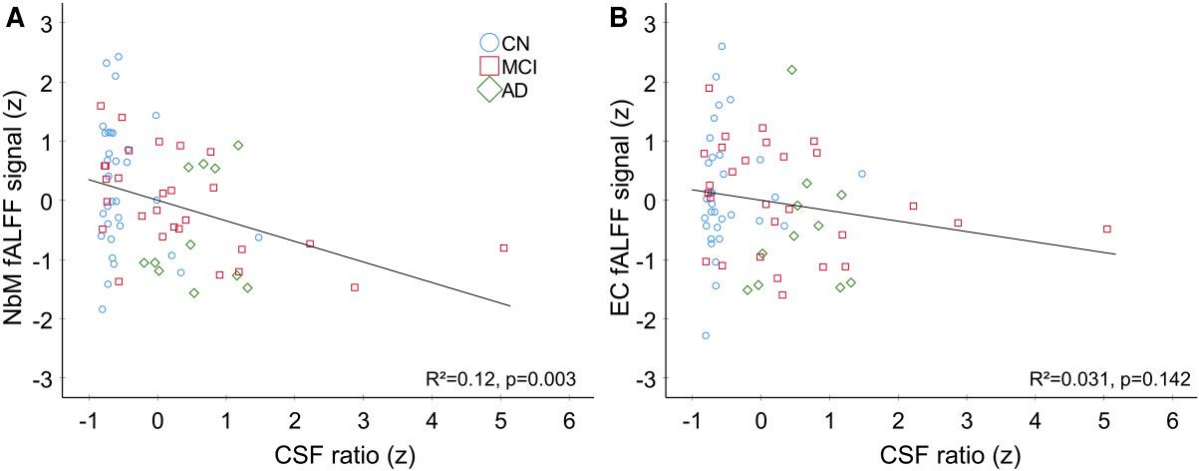
**Linear regression for *Z*-scored fALFF signal at baseline (*t*_1_) against *Z*-scored CSF ratio for nucleus basalis of Meynert (NbM) (A) and entorhinal cortex (EC) (B).** For the sake of visualization, the groups are plotted in different colours and shapes representing individual data points: blue circle for CN (*n* = 32), red square for MCI (*n* = 28) and green rhombus for AD (*n* = 11). The linear regression was significant only in the NbM (**A**) [*R*²=0.120, *F*(1, 69) = 9.437, *P* = 0.003] but not EC (**B**) [*R*²=0.031, *F*(1, 69) = 2.206, *P* = 0.142], indicating a region-specific decrease in functional activity and proteinopathology.

In a next step, we used linear regressions on baseline fALFF values from NbM and EC, respectively, with CSF ratio as independent variable. It revealed a significant linear effect in the NbM [*R*²=0.12, *F*(1, 69) = 9.437, *P* = 0.003; Fig. 2A] but not EC [*R*²=0.031, *F*(1, 69) = 2.206, *P* = 0.142; Fig. 2B]. A direct comparison of both correlations (NbM versus EC, one-tailed, which was justified by our a priori hypotheses) revealed a significant difference that was driven by a more negative correlation in NbM compared with EC (*Z* = −1.94; *P* = 0.0262; 95% CI: −0.3429 to 0.0015). Additionally, we analysed both linear regressions independently for Aβ and pTau (Supplementary Fig. 1). It revealed significant effects in NbM [pTau: *R*²=0.057, *F*(1, 69) = 4.19, *P* = 0.044; Supplementary Fig. 1A; Aβ: *R*²=0.09, *F*(1, 69) = 6.784, *P* = 0.011; Supplementary Fig. 1C] but not in EC [pTau: *R*²=0.022, *F*(1, 69) = 1.556, *P* = 0.217; Supplementary Fig. 1B; Aβ: *R*²=0.003, *F*(1, 69) = 0.234, *P* = 0.63; Supplementary Fig. 1D].

### Baseline signal in NbM predicts APSC in EC fALFF

**Figure 3:**
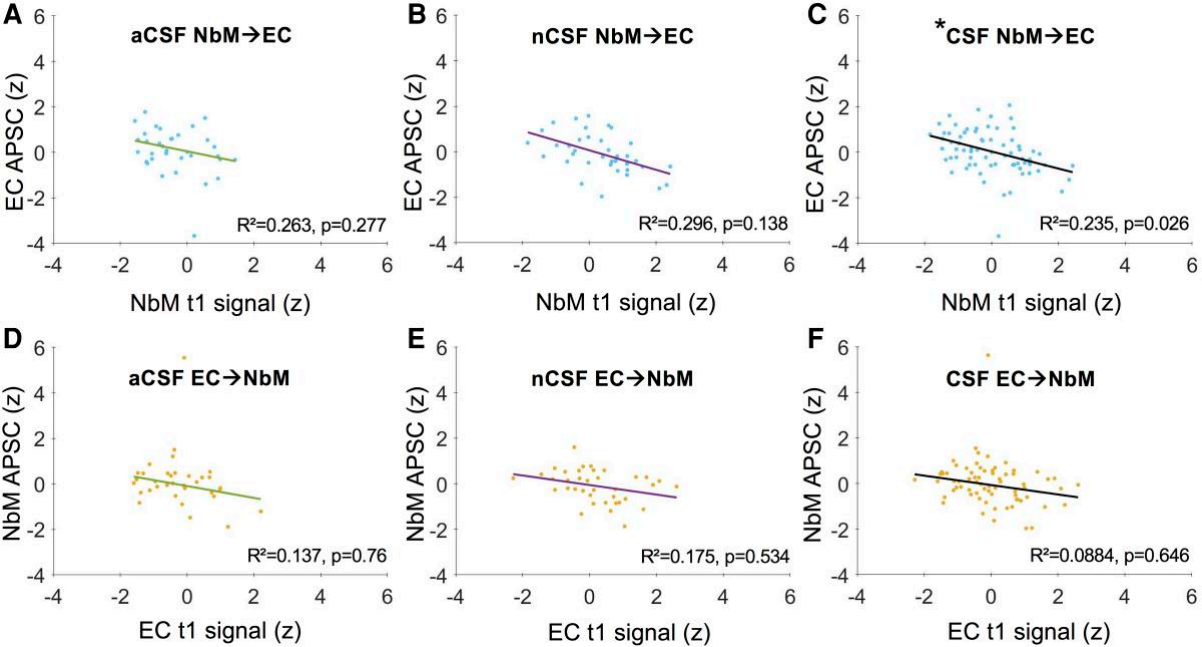
**Baseline signal in the nucleus basalis of Meynert (NbM) predicts APSC in the entorhinal cortex (EC) fALFF**. Plots for robust regression models for NbM→EC (**A**, **B**, **C**) and EC→NbM (**D**, **E**, **F**). The *x*-axis indicates *Z*-scores for baseline (*t*_1_) signal, and the *y*-axis indicates *Z*-scored APSC. There was a significant effect for NbM→EC [**C**, *R*²=0.235, *F*(8, 62) = 2.39, *P* = 0.026], with NbM as a significant predictor of EC’s APSC [*r* = −0.3751, *t*(62)=−3.1445, *P* = 0.003]. There was no significant effect for EC→NbM [*R*²=0.0884, *F*(8, 62) = 0.751, *P* = 0.646]. Each data point represents an individual value. aCSF, abnormal CSF (*n* = 34); nCSF, normal CSF (*n* = 37); CSF, CSF normal and abnormal included as a variable (*n* = 71). *P < 0.05.

To further address the temporal changes in Alzheimer’s disease progression, we examined whether the baseline signal in one region predicts the APSC in the other region. Here, in a first step, we used robust regression modelling for both competing models separately for normal CSF versus abnormal CSF. They revealed no significant effect for NbM→EC in abnormal CSF [*R*²=0.263, *F*(7, 26) = 1.33, *P* = 0.277; Fig. 3A; Supplementary Table 1] and no significant effect for NbM→EC in normal CSF [*R*²=0.296, *F*(7, 29) = 1.74, *P* = 0.138; Fig. 3B; Supplementary Table 1]. Similarly, there was no significant effect for EC→NbM in abnormal CSF [*R*²=0.137, *F*(7, 26) = 0.587, *P* = 0.76; Fig. 3D; Supplementary Table 1] and no significant effect for EC→NbM in normal CSF [*R*²=0.175, *F*(7, 29) = 0.88, *P* = 0.534; Fig. 3E; Supplementary Table 1].

In a second step, we analysed both groups together by including CSF group in the two competing regression models. Importantly, we observed a statistically significant effect for the model NbM→EC [*R*²=0.235, *F*(8, 62) = 2.39, *P* = 0.026; Fig. 3C; Supplementary Table 1], with NbM as a significant predictor of EC’s APSC [*r* = −0.3751, *t*(62)=−3.1445, *P* = 0.003, confidence interval (CI): −0.6136 to −0.1366]. The other regression model EC→NbM did not show a significant effect [*R*² = 0.0884, *F*(8, 62) = 0.751, *P* = 0.646; Fig. 3F; Supplementary Table 1]. Replacing CSF as dichotomous predictor by the continuous CSF ratio did not change the results (i.e. significant effects for the model NbM→EC, *P* = 0.021, but not EC→NbM, *P* = 0.466).

### CSF group does not moderate the relationship of NbM and EC in fALFF

Finally, we performed two moderation analyses. The first model included baseline fALFF NbM as independent variable, fALFF EC APSC as dependent variable and CSF group as moderator. The model was statistically significant [*R*²=0.2215, *F*(9, 61) = 3.4009, *P* = 0.0019], with a significant direct effect of NbM→EC [*t*(61)=−3.442, *P* = 0.001], but no significant moderator effect [*t*(61) = 0.4095, *P* = 0.6836], which is in line with the robust regression analysis.

The second model included baseline fALFF EC as independent variable, fALFF NbM APSC as dependent variable and CSF group as moderator. The model was not statistically significant [*R*²=0.0965, *F*(9, 61) = 0.7857, *P* = 0.6303], which, again, is in line with the robust regression analysis.

The results for ReHo (Supplementary Figs. 2A, B and 3; Supplementary Table 2) and functional connectivity (Supplementary Fig. 2C) can be found in the Supplementary material.

## Discussion

We investigated the functional properties of the human NbM and EC in relation to the disease progression of Alzheimer’s disease based on longitudinal rsfMRI data and CSF markers of Aβ and pTau. With a focus on fALFF, our data provide evidence that spontaneous local brain activity in the NbM, but not EC, is reduced with CSF ratio, and, importantly, it predicts the APSC in the interconnected EC independently from proteinopathology. As such, our findings extend previous anatomical studies in humans and animals by providing novel physiological insights into the pathological staging model of Alzheimer’s disease suggesting the NbM as an early origin for subsequently affected brain regions possibly via a trans-synaptic mechanism.

Local spontaneous brain activity, as quantified by fALFF, was reduced in the NbM at baseline in the abnormal CSF group (Fig. 1B), and there was a linear reduction in fALFF activity with CSF ratio (Fig. 2A). Importantly, both relationships were only observed in the NbM but not in the EC (Fig. 2B), which further underlines that the NbM is specifically vulnerable to Alzheimer’s disease progression. In fact, pTau and Aβ are two proteins that have been associated with Alzheimer’s disease^35^ and the NbM is particularly vulnerable to the early accumulation of pTau^36-38^ and Aβ deposition.^39^ This may be due to the fact that cholinergic basal forebrain neurons have rather large axons and arbors reaching into the entire central nervous system with high metabolic demands for maintenance, reparation and transportation.^40^ At the same time, simply due to their sizes, they are more vulnerable to toxins,^41^ which may further promote disease progression.

The pathological staging model suggests a structural degeneration spreading from the NbM to the EC, which adds a crucial upstream link to Alzheimer’s degeneration.^1^ Our functional data support such a view by showing that the NbM’s baseline fALFF signal predicted the APSC in the EC (Fig. 3C) but not the reverse (Fig. 3F). Interestingly, this effect was independent of CSF status, which was further supported by the absence of a moderating effect of CSF. While this is compatible with a specific spread from NbM to EC, it also indicates that the putative functional consequences, namely changes in neural activation, are unrelated to pTau and Aβ. This apparently differs from anatomical changes from NbM to EC that were more pronounced in subjects with abnormal CSF.^1^ From a physiological point of view, a trans-synaptic spread of proteins between anatomically interconnected brain regions is possible and has been shown in several animal studies. For instance, aggregates of tau can propagate from the EC to other limbic regions, including the dentate gyrus and hippocampal CA fields, followed by neocortical brain regions including the parietal cortex.^3-5,42^  *In vitro*, this can be enhanced by neural activity,^6^ which might help to explain why CSF status did not moderate the relationship between NbM activity and longitudinal changes in EC activity in our study. While this needs to be further investigated using larger and independent samples, our study is the first to show *in vivo* in humans that a neural signal in NbM can serve as a predictive marker for functional changes in the anatomically interconnected EC across healthy controls, MCIs and Alzheimer’s disease patients.

Although fALFF is a prominent marker of spontaneous local brain activity,^11,12^ only a limited number of studies used fALFF to investigate Alzheimer’s disease. Importantly, previous work did not specifically focus on the NbM and EC but other, typically larger, brain regions. It showed, for instance, decreased fALFF signals in the bilateral middle frontal and left precuneus in participants with positive Aβ.^14^ In preclinical Alzheimer’s disease, increases and decreases in fALFF were reported in the right inferior frontal gyrus,^14,43^ and in prodromal Alzheimer’s disease, lower fALFF signals could be shown in the bilateral precuneus, right middle frontal gyrus, right precentral gyrus and postcentral gyrus. Finally, in Alzheimer’s disease, fALFF was increased in the right fusiform gyrus, medial temporal lobe and inferior temporal gyrus but decreased in the bilateral precuneus, left posterior cingulate cortex, left cuneus and superior occipital gyrus.^43^ These partly divergent effects of fALFF associated with Alzheimer’s disease might be explained by compensatory effects to maintain an adequate level of cognitive performance^43^ and could be a functional hallmark of neural aging^44^ that needs further attention. Furthermore, since no significant effects in ReHo and functional connectivity were detected (see Supplementary material), fALFF seems to be a particularly sensitive marker. Together, fALFF is highly sensitive to changes in neural activity associated with Alzheimer’s disease even in rather small brain regions and therefore offers a useful marker in future studies.

Our analyses specifically focused on the functional properties of the human NbM and EC but no other interconnected brain regions that, according to the pathological staging model, follow the EC. These may include the parahippocampal cortex and hippocampal structures, as well as the parietal cortex.^1,3-5,42^ Along these lines, we included functional signals averaged from both hemispheres, which simplified our analyses, but it neglected possible lateralization effects.^45,46^ The NbM is most likely not the source of Alzheimer’s disease pathology. In fact, post-mortem histology revealed the locus coeruleus as highly vulnerable to early degeneration even before the NbM.^47,48^ Here, we did not include the locus coeruleus in our analyses since the rsfMRI data did not provide the necessary special resolution to extract a reliable signal from this rather small brain region.^49^

Finally, ADNI is a large multicentre study offering a rich and unique data set. This also means that our rsfMRI data come from different MR scanners, possibly leading to a bias in image quality and extracted signal. Therefore, we only included high-quality data based on comparable protocols and within-subject measurements from the same scanner. We also employed appropriate covariates in our statistical models, and differences in scanning parameters (e.g. slice order or number of volumes) were accounted for during preprocessing.^50,51^ Further, our main findings are based on analyses including a measure of APSC, which is robust against within-subject variability, e.g. because of the MR scanner. Along these lines, another possibility of analysing our data would be based on the AT(N) framework, which includes markers of Aβ, tau (T) and neurodegeneration (N).^24^ However, this requires a much larger sample of subjects than *n* = 71, since at least three groups would need to be included (A−T−, A + T−, A + T+), and their distribution is typically unbalanced (see, e.g. Zeng *et al*. ^52^). Furthermore, the necessary cut-off values, which are often based on PET but in some studies CSF, are not well defined.^24^ Finally, we wanted to analyse our data as similar as possible to Fernández-Cabello *et al*. (2020) in order to be comparable.

## Conclusions

Functional activity in the human basal forebrain scaled with proteinopathology and predicted the functional decline within the interconnected EC independent from CSF status. As such, our findings extend the pathological staging model of Alzheimer’s disease by giving novel insights into the functional properties of the underlying brain regions. From a more general perspective, fALFF appears to be a suitable marker to further investigate functional brain changes associated with the progression of Alzheimer’s disease.

## Supplementary Material

fcad262_Supplementary_Data

## Data Availability

All data are freely available upon request from the IDA run by the LONI (https://ida.loni.usc.edu). The data that support the findings of this study are available upon reasonable request from the corresponding author.
